# Epidural Electrical Stimulation: A Review of Plasticity Mechanisms That Are Hypothesized to Underlie Enhanced Recovery From Spinal Cord Injury With Stimulation

**DOI:** 10.3389/fnmol.2020.00163

**Published:** 2020-09-02

**Authors:** Jaclyn T. Eisdorfer, Rupert D. Smit, Kathleen M. Keefe, Michel A. Lemay, George M. Smith, Andrew J. Spence

**Affiliations:** ^1^Department of Bioengineering, College of Engineering, Temple University, Philadelphia, PA, United States; ^2^Department of Neuroscience, Shriners Hospitals Pediatric Research Center, Lewis Katz School of Medicine, Temple University, Philadelphia, PA, United States

**Keywords:** plasticity, electrical epidural stimulation, propriospinal detours, monosynaptic connections, internal motor copy, efferent motor copy, designer receptor exclusively activated by designer drugs (DREADDs), afferent stimulation

## Abstract

Spinal cord injury (SCI) often results in life-long sensorimotor impairment. Spontaneous recovery from SCI is limited, as supraspinal fibers cannot spontaneously regenerate to form functional networks below the level of injury. Despite this, animal models and humans exhibit many motor behaviors indicative of recovery when electrical stimulation is applied epidurally to the dorsal aspect of the lumbar spinal cord. In 1976, epidural stimulation was introduced to alleviate spasticity in Multiple Sclerosis. Since then, epidural electrical stimulation (EES) has been demonstrated to improve voluntary mobility across the knee and/or ankle in several SCI patients, highlighting its utility in enhancing motor activation. The mechanisms that EES induces to drive these improvements in sensorimotor function remain largely unknown. In this review, we discuss several sensorimotor plasticity mechanisms that we hypothesize may enable epidural stimulation to promote recovery, including changes in local lumbar circuitry, propriospinal interneurons, and the internal model. Finally, we discuss genetic tools for afferent modulation as an emerging method to facilitate the search for the mechanisms of action.

## Introduction

Spinal cord injury (SCI) often results in life-long sensorimotor dysfunction. Although regeneration within the adult spinal cord is limited, some spontaneous or activity-dependent sensorimotor recovery still occurs, mostly mediated by localized sprouting and plasticity of axon terminals (Waters et al., 1996; Burns et al., 1997). Substantial recovery after trauma is challenging because of the poor ability of supraspinal axons to regenerate and form functional networks below the level of injury. The loss of these vital inputs reduces the generation, regulation, and patterning of motor outputs. Improvements in motor function can be achieved with locomotor training, rehabilitation, and/or increased neuronal activity.

These methods activate axonal growth pathways (e.g., GAP43; Storer and Houle, 2003) to enhance sprouting and plasticity to either establish circuits that bypass the lesion to relay motor commands and/or increase connections onto vital motor circuits. Over the years, direct electrical stimulation of cortical or supraspinal neurons demonstrated that activity plays an important role in mediating plasticity induced sensorimotor recovery (Martin, 2016). More recently, stimulation of spinal sensory axons with electrodes placed epidurally has shown benefits in promoting functional recovery. Indeed, epidurally placed electrodes can stimulate afferents in specific patterns to increase the excitability of networks to drive voluntary and autonomically controlled motor responses (Edgerton and Harkema, 2011). Although multiple mechanisms have been proposed, the neuroplastic changes that underlie these improvements are not yet well understood. A better understanding of these mechanisms or circuits would be beneficial in the development of combined therapies to augment sensorimotor improvements using epidural stimulation to further enhance the recovery of sensorimotor function in individuals with SCI.

The foundation of artificially modulating neurons with electrical stimulation was borne from the search for pain management. In the first century AD, Scribonius Largus, a Roman physician, reportedly advised patients to sit in pools of water electrified by torpedo fish to numb distal extremity pain (Moller, 1995). It was not until 1967, however, that epidural stimulation was first used and approved by the FDA for suppression of intractable pain (Shealy et al., 1967). Then, in 1976, epidural electrical stimulation (EES) was introduced to alleviate spasticity due to Multiple Sclerosis, and it was anecdotally noticed that patients improved in motor function (Cook, 1976). EES was also identified to reduce spasticity (Barolat et al., 1988) and allow for voluntary mobility across the knee or ankle in several SCI patients, further indicating its utility in supplementing motor activation (Dimitrijevic et al., 1986).

Activity-based training in conjunction with EES can bolster use-dependent plastic changes in sensorimotor circuits caudal to the injury site (Courtine et al., 2009). In a seminal paper by Harkema et al. (2011), it was demonstrated in humans that EES can enhance weight-bearing standing, stepping, and volitional movement of leg muscles when in a supine position. This work was followed up with similar demonstrations in individuals with motor complete paralysis for intentional control of movements of the lower limbs (Angeli et al., 2014; Grahn et al., 2017) as well as independent stepping during EES activation (Gill et al., 2018). Similarly, in clinical studies, central and peripheral electrical stimulation improved sensorimotor function (Guiraud et al., 2014; Possover, 2014), such as weight-bearing, standing (Crosbie et al., 2014), and walking (Herman et al., 2002; Hardin et al., 2007; Karimi et al., 2013; Possover, 2014). Emerging evidence suggests that closed-loop and/or phasic EES is more efficacious in promoting functional recovery in humans than tonic stimuli. Unlike closed-loop and phasic stimuli, continuous input increases the probability of antidromic collisions in proprioceptive afferents, thereby disrupting sensory information, especially in humans, as they have longer nerves. As such, stimulation protocols restricted to a range of frequencies and amplitudes appear to better facilitate recovery and locomotion (Formento et al., 2018).

In addition to enhanced sensorimotor recovery, EES can improve cardiovascular (Harkema et al., 2018; West et al., 2018), autonomic (Gad et al., 2014, 2018), and respiratory (Hachmann et al., 2017) functions as well as body weight composition (Terson de Paleville et al., 2019) in individuals with motor complete paralysis. Despite relatively small sample sizes, EES has shown remarkable therapeutic potential as an intervention for SCI. However, the mechanisms that underlie EES-induced long-term recovery remain elusive. It is widely believed that EES activates large and medium diameter afferents within the posterior roots in humans and animals (Murg et al., 2000; Rattay et al., 2000; Courtine et al., 2009; Capogrosso et al., 2013). Indeed, computational modeling studies corroborated with electrophysiological and pharmacological data of afferent populations indicate specifically that group Ia/Ib/II proprioceptive and low-threshold cutaneous afferents are all affected by electrical stimulation (Bouyer and Rossignol, 1998; Rossignol et al., 2006; Capogrosso et al., 2016). Recent data suggest that proprioceptive input has the greatest influence on circuit reorganization during recovery and that the ablation of proprioceptors permanently reverts sensorimotor improvements to the injured state (Capogrosso et al., 2013; Takeoka et al., 2014; Takeoka and Arber, 2019; Takeoka, 2020). Congruently, Formento et al. (2018) proposed that if the chosen EES stimuli block proprioceptive input, individuals with SCI are unable to show meaningful locomotor improvements.

Here we explore three endogenous mechanisms of sensorimotor plasticity by which EES may induce locomotor recovery through stimulation of peripheral proprioceptive afferents: direct strengthening of monosynaptic connections; dynamic reorganization of Propriospinal neurons (PNs) around and below the lesion site; and the influence of the internal models for error correction and learning proper patterning (*via* interneurons). These mechanisms would likely behave synergistically, integrating, and functioning in concert to promote recovery. In this review, we discuss these mechanisms and their putative roles in supporting sensorimotor improvements after SCI and consider how molecular tools for afferent modulation can accelerate uncovering the changes in circuitry that drive recovery.

## Plasticity Mechanisms That Are Hypothesized to Enable EES to Promote Enhanced Locomotor Recovery After SCI

### Hypothesis 1: Strengthening of Monosynaptic Connections Between Proprioceptive Afferents and Motorneurons

**Figure 1:**
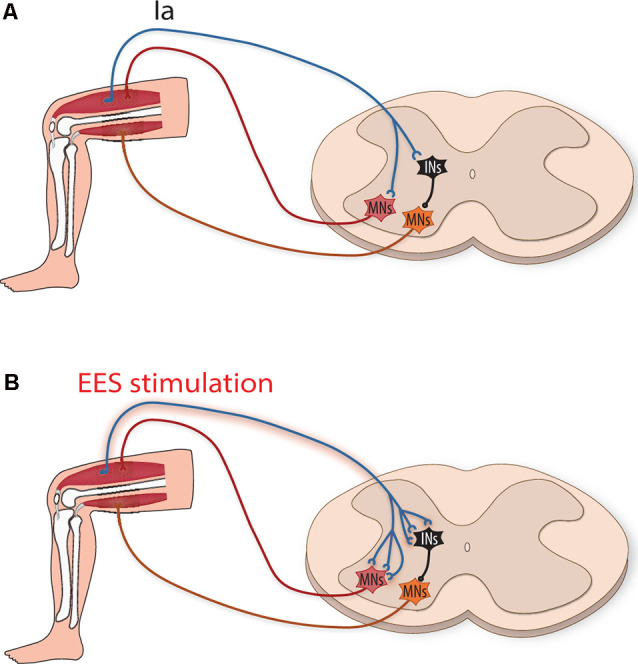
Strengthening of monosynaptic connections by epidural electrical stimulation (EES) induced activation of group Ia afferents. **(A)** Schematic illustration depicting group Ia afferents directly activating motoneurons (MNs, red) to activate agonistic muscles and indirectly (orange MNs) to inhibit antagonistic muscles (*via* inhibitory interneurons, iINs, black). **(B)** After spinal cord injury (SCI), EES increases the activity of type Ia afferents, putatively strengthening their connections by inducing sprouting and new synapse formation onto MNs and iINs.

Perhaps the most straightforward form of plasticity for enhancing motor output with EES after SCI is strengthened connections between stimulated afferents and motoneurons that reside in nearby lumbar spinal cord segments (Figure 1). Within sensory afferent populations, proprioceptive neurons provide information concerning muscle length, velocity, and force development that are thought to be used to estimate limb position and other aspects of movement dynamics. Within the spinal cord cutaneous and proprioceptive axons branch extensively, relaying limb positional information and force dynamics to multiple spinal cord levels, and supraspinal and somatosensory cortical regions. Of these sensory afferents, group Ia proprioceptive axons establish direct monosynaptic connections onto motoneurons that innervate agonist muscles as well as interneuronal circuits within motor pools. Both of these circuits involving proprioceptive afferents thought to be critical for locomotor recovery after SCI (for a recent review see Takeoka, 2020). Animal models lacking muscle spindle feedback (Takeoka et al., 2014) or after the loss of proprioceptive afferents (Takeoka and Arber, 2019) fail to regain control of affected hindlimbs and inappropriately reorganize descending circuitry (Takeoka, 2020). Proprioceptive ablation following recovery from SCI also permanently regresses sensorimotor improvements to the injured state (Takeoka and Arber, 2019). While EES can activate large and medium diameter afferents of the dorsal roots, proprioceptive afferents have been proposed to be the most influential in regaining volitional control of affected muscles.

During activity, EES is thought to work by boosting muscle recruitment *via* the activation of Ia muscle spindle afferents (Moraud et al., 2016). Although this procedure works well in animal models, the length of peripheral nerves in humans makes it less effective by increasing the probability of antidromic collisions, thereby reducing the propagation of naturally occurring proprioceptive action potentials (Retamal et al., 2018). Recent forms of EES that employ spatiotemporal modulation (Wenger et al., 2016; Wagner et al., 2018) show improvement in human locomotion because they activate appropriate muscles (*via* spatial localization within the cord—flexors vs. extensors, hip vs. ankle, etc.) in concordance to swing-stance rhythmicity without negatively effecting endogenous proprioceptive information.

Activity-dependent stimulation can strengthen connections between neurons by enhancing the efficacy of existing synapses (Davis et al., 1985), as well as by inducing growth-promoting factors that enhance axonal sprouting and result in the formation of new synapses (Retamal et al., 2018; Xu et al., 2019). Whether these mechanisms occur between group Ia afferents and motoneurons (Figure 1) is an open question (Wolpaw and Lee, 1989). If they do occur, it would enhance group Ia afferent drive of motoneurons and possibly the motoneuron drive (Heckman and Enoka, 2012). This, in turn, would enhance muscle activity by supplementing activity provided from partially denervated motor subsystems that, after SCI, contribute insufficient locomotor drive. Interestingly, although the majority of Ia connections onto motoneurons occur within the same muscle target, they also establish a lower number of connections onto functionally related muscles (Eccles et al., 1957). Thus, sprouting of group Ia afferents onto these muscle synergists could increase the activation of several muscles within a particular extensor or flexor group, thereby increasing the overall force generated.

Not only do proprioceptive group Ia afferents activate the agonist muscle (Mears and Frank, 1997), they also indirectly inhibit the antagonist muscles *via* inhibitory neurons (Hultborn et al., 1971; Figure 1). EES facilitation or sprouting of additional synapse formation of group Ia afferents onto inhibitory interneurons could help facilitate locomotion by supporting stronger inhibition of antagonist muscle activity at appropriate phases of movement. Whether local plastic changes in proprioceptors such as these can influence helpful rearrangement of descending pathways is unknown (Lamy et al., 2010), but mice with genetic ablation of these proprioceptors are unable to form these functional reorganizations (Takeoka et al., 2014; Takeoka, 2020).

### Hypothesis 2: Reorganization of Propriospinal Circuitry Around the Lesion Site and Within the Lumbar Central Pattern Generator to Promote Rhythmic Activity and Hindlimb Coordination

PNs play a crucial role in locomotion by integrating sensory and motor information to coordinate multiple muscle groups. Functionally they may work to achieve tasks such as maintenance of balance and may be part of the neural substrate that results in “motor synergies,” acting to, e.g., adjust the dynamics of synergistic muscles after perturbation (Miller and Van der Burg, 1973; Levine et al., 2014). For this review, we use the definition of a PN as proposed by Flynn et al. (2011): a neuron whose soma is located within a spinal segment and whose axons project ipsilaterally and/or contralaterally to a different spinal segment and/or to supraspinal centers. Anatomically, PNs can be classified as “short” if their projections span less than seven spinal segments, including commissural interneurons and several genetically defined interneuronal types, and “long” if they span seven or more spinal segments (Conta and Stelzner, 2009; Flynn et al., 2011). PN circuits are modulated by descending input from supraspinal pathways (e.g., information containing motor commands) and/or sensory input from peripheral afferents (Cowley and Schmidt, 1997; Levine et al., 2014).

Modeling and experimental studies have demonstrated that PNs (short and long) may be an important CPG supraspinal target for the control of locomotion and fore-hind coordination (Ballion et al., 2001; Danner et al., 2017; Ausborn et al., 2019; Lin et al., 2019; for reviews see Flynn et al., 2011; Laliberte et al., 2019). In its traditional formulation, the vertebrate locomotor CPGs (one CPG each per hindlimb) are located within the spinal cord, and each consists of a “half-center” oscillator where flexors and extensors mutually inhibit each other (Brown, 1914). Current versions have the half-centers organized into two-levels—rhythm generator and pattern formation networks—which are both susceptible to supraspinal and peripheral afferent modulation during locomotion, but can also generate rhythmic behavior in the absence of these feedbacks (Brown, 1911; Rybak et al., 2006a,b). Interactions between these half-centers are coordinated by the activities of short (commissural, V01, etc.) and long PNs under the control of the supraspinal centers (Rybak et al., 2006a,b, 2015; Cowley et al., 2008, 2010; Zaporozhets et al., 2011).

Contained within the spinal cord, PNs are well-suited to relay information to motor pools below a lesion site (Han et al., 2019). Many receive inputs from supraspinal motor systems, and after unilateral lesion, corticospinal tract (CST) or reticulospinal (ReST) tract axons can sprout onto cervical PNs to relay these motor commands past the lesion site (Bareyre et al., 2004; Filli et al., 2014). After injury PNs upregulate GAP-43, neurotrophic factors, tubulins, and neurofilaments, all of which contribute to elongation and axonal sprouting (Fernandes et al., 1999; Siebert et al., 2010; Taccola et al., 2018; Wang et al., 2018). Indeed, 8 weeks after unilateral thoracic hemisection, long descending PNs bypassing the lesion undergo distal sprouting and show a doubling of connectivity onto lumbosacral motoneurons (Bareyre et al., 2004). Reorganization of PN networks is 2-fold: the circumnavigation of the injury site and plasticity below the level of injury. Delayed staggered hemisection studies demonstrated the ability of PNs to detour around the lesion to provide a surrogate flow of supraspinal locomotor commands to motor pools below the level of injury (Kato et al., 1984; Courtine et al., 2008; May et al., 2017). Propagation of these locomotor commands through PNs can elicit the rhythmic activity of motoneurons of the lumbar CPG (Cowley et al., 2008). Detouring lesions cannot occur in a complete SCI, however, animal models often exhibit some sensorimotor recovery. This is due to the plasticity of the PN network below the level of injury (Howland et al., 1995; Fenrich and Rose, 2009; Laliberte et al., 2019). Even after disrupting the flow of supraspinal motor commands, exogenously-augmented changes in PN circuitry can lead to the re-emergence of locomotion. In multiple animal models, PN networks induce locomotor-like activity in the absence of supraspinal input as shown in, for example, an *ex vivo* preparation of the spinal cord with drug administration (Zaporozhets et al., 2011) and after complete spinal cord transection with electrical stimulation (Yakovenko et al., 2007). Thus, these interneuronal networks can adapt to the loss of supraspinal input *via* dynamic reorganization, and can partially compensate for the loss of higher-level control if their activity is directly or indirectly bolstered by an exogenous source.

**Figure 2:**
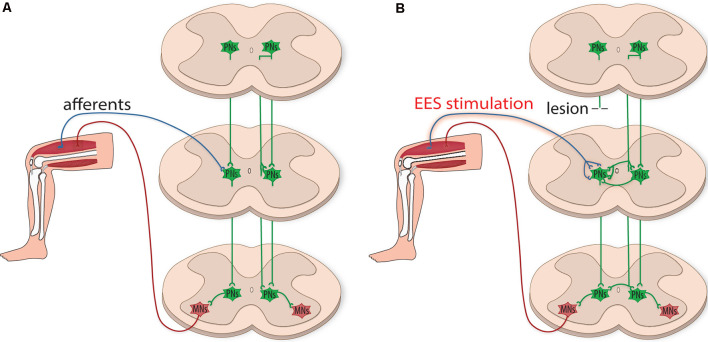
Plasticity of propriospinal neuronal circuitry after injury. **(A)** Schematic illustration depicting the integration of sensory and motor information by propriospinal neurons (PNs, green). PNs project ipsilaterally and contralaterally onto motoneuron pools (MNs, red) and contribute to interlimb coordination by integrating information relayed from supraspinal centers and received from sensory input. **(B)** After the injury, PNs have the plastic potential for dynamic reorganization. EES can indirectly recruit and modulate propriospinal circuits, creating functional networks caudal to the lesion site. PNs can relay supraspinal input by circumventing the injury, providing a surrogate flow of supraspinal locomotor commands to MNs. PNs can also reorganize below the level of injury, sprouting contralaterally to facilitate communication across multiple spinal segments.

Although EES does not directly target PNs, evidence suggests EES can indirectly recruit and modulate these circuits, through the activation of peripheral sensory afferents, to facilitate hindlimb stepping (Capogrosso et al., 2013; Moraud et al., 2016; Formento et al., 2018). Notably, enhanced proprioceptive input provides critical guidance to organize the plasticity of PNs to circumvent a lesion site and relay information below the level of injury (Courtine et al., 2008; Takeoka and Arber, 2019). Also, Hebbian-like processes directed by electrically-enhanced sensory afferents and spared supraspinal projections could strengthen terminal contacts of PNs within motor pools in the lumbar CPG, which may be susceptible to Hebbian facilitation (Righetti et al., 2006). Even though spared supraspinal projections provide insufficient drive to activate locomotion, the additional drive provided by PN bypass relays could enhance supraspinal control to promote regain of function in animal models with severe SCI (Courtine et al., 2008). Spared PN circuitry, which can remain dormant after injury, may also play a vital role in this relay mechanism. Indeed, recovery of some volitional control in chronically paralyzed patients (Harkema et al., 2011; Angeli et al., 2014) may be a consequence of reactivating dormant spared PN circuitry indirectly *via* EES. Prolonged electrical stimulation may also promote propriospinal neuronal sprouting, which can strengthen newly formed and spared connections (Figure 2).

Several genetically identified PNs may play distinct roles in locomotor recovery in part by propagating locomotor commands to the lumbar CPGs (Laliberte et al., 2019). V1 PNs are inhibitory interneurons that project ipsilaterally onto motoneurons, as well as onto other V1 PNs and inhibitory interneurons. V1 PNs putatively inhibit motoneurons that innervate flexor muscles for the facilitation of coordination of flexor and extensor activity (Alvarez et al., 2005). Likewise, V2b PNs coordinate flexor and extensor activity, but possibly do so *via* inhibition of extensor muscles (Britz et al., 2015). V2a PNs, however, act as excitatory messengers to commissural interneurons for left-right coordination. For example, Dougherty and Kiehn (2010) proposed that a subpopulation of nonrhythmic V2a interneurons mediate sensory-evoked locomotor-like activity by being recruited at different speeds to help regulate right-left coordination and ipsilateral firing of motoneurons. V3 PNs are also excitatory interneurons, but function to stabilize ipsilateral and contralateral patterns of locomotion. Further, during postmitotic development, V3 interneurons migrate dorsally or ventrally and develop distinct functions: dorsal V3 interneurons receive robust input from group Ia proprioceptive neurons and might be indirectly involved in adjusting right-left coordination, whereas ventral V3 interneurons were suggested to synchronize motor output amongst multiple motoneuron pools (Borowska et al., 2013; Lin et al., 2019). This work was followed with a computational model of the locomotor CPG demonstrating that as speed increases, sensory afferents relay limb speed onto V3 interneurons, with V3 interneurons assisting in the transition from alternating to synchronized gaits (Danner et al., 2017). dI3 PNs also receive sensory information from the periphery and directly activate motoneuron pools in the cervical and lumbar CPGs driving ipsilateral agonist muscles (Bui et al., 2013, 2016). Importantly, dI3 PNs have been identified to promote rhythmic locomotor recovery after SCI even in the absence of supraspinal input, suggesting an essential role of these PNs in the transmission of activity between adjacent spinal segments that contain lumbar CPG components (Bui et al., 2016). Together, plasticity among different types of PNs could influence locomotion by enhancing supraspinal drive through relays bypassing the lesion as well as supporting rhythm generation to increase stepping patterning.

As CPGs may be sensitive to Hebbian facilitation (Righetti et al., 2006), it is the convergence of activity (e.g., peripheral afferents with increased activity from EES, PN networks, and spared supraspinal projections) within lumbar motor pools that is likely responsible for driving locomotor recovery after SCI (Dimitrijevic et al., 1998; Guertin, 2013). Ultimately, it is the activation of the lumbar CPGs that may facilitate improvements in individuals with incomplete SCI (Herman et al., 2002) and generate stepping-like movements *via* tonic input in individuals with complete SCI (Minassian et al., 2004, 2007). Importantly, peripheral afferent activation from EES can modulate the lumbar CPGs to adapt to perturbations and entrain it appropriately to drive recovery (Young, 2015). However, studies with split-belt locomotion suggest that this phenomenon results from side-specific proprioceptive input and PNs are necessary to transfer information to contralateral sides of the spinal cord (Prokop et al., 1995). As such, PNs are not only recruited by EES, particularly those that are susceptible to afferent input (e.g., V3 and dI3 PNs) but perhaps are also required for the transmission of rhythmic activity throughout the lumbar CPGs to elicit hindlimb coordination.

### Hypothesis 3: Spatiotemporal Integration of the Internal Model With Peripheral Afferent Input Within Interneuronal Networks to Aid Learning of Correct Motor Output

Motor activity requires precise timing to coordinate a series of individual muscle contractions in sequence so that the movement can proceed smoothly. Disruption of descending motor control pathways reduces vital input into spinal motor systems reducing coordination and inducing movement errors. Here, we discuss circuits known to influence the timing of muscle contraction, error correction, motor learning, and movement patterning as possible mechanisms by which increased afferent activation could enhance recovery.

Error correction and motor memory have been studied extensively within cerebellar circuits. One such circuit is the internal forward dynamic model; derived from internally generated motor signals, this circuit is used to predict the motor and sensory consequences of an action (Wolpert et al., 1995; Wolpert and Ghahramani, 2000; Bui et al., 2013). These predictions are then compared with actual sensory data to either identify errors in the motor program or possible external perturbations of the limb. Prediction calculations are primarily performed in the cerebellum from planned motor commands driving a forward model of the limb. Simultaneously, proprioceptive and low-threshold cutaneous information is transmitted to the cerebellum (*via* the dorsal and ventral spinocerebellar tracts respectively), where comparative analysis of the incoming information is processed and directed back to the spinal cord through the ReST. Anatomically, the ventral spinocerebellar pathway is also responsible for carrying a spinal copy of motor commands of rhythmic activity (e.g., locomotion) back to the cerebellum (Brownstone et al., 2015). The reticulospinal tract extends from the caudal midbrain through the pons and medulla with its axons descending *via* the ventrolateral funiculus of the spinal cord, eventually forming glutamatergic synapses with spinal interneurons and primary motoneurons (Brownstone and Chopek, 2018). With the repetition of the task, discrepancies in the motor program are eliminated to generate a progressively more refined motor memory (Tuthill and Azim, 2018).

When discussing the internal model, which is composed of the inverse model, forward model, and efferent copy, it is important to define the pathways involved. The inverse model determines the motor commands necessary to achieve the desired movement, where the inputs are the desired state of a limb, and the outputs are the motor commands needed to achieve that state. The forward model simulates the forward dynamics of the limb given a set of motor commands and produces an estimate of the final state (motor and sensory) of the limb; the inputs are the commands issued by the central nervous system, and the outputs are the predicted limb outcomes (Wolpert and Miall, 1996; Kawato, 1999). The efference copy is a copy of the motor command delivered to the muscles and can be used as input to the forward model to predict expected motor output and sensory feedback (Kawato, 1999). For this review, we refer to the entirety of this endogenous system (including the ReST) as the descending supraspinal control, and we propose that it is a contributory mechanism involved in recovery from SCI both with and without spinal cord stimulation.

**Figure 3:**
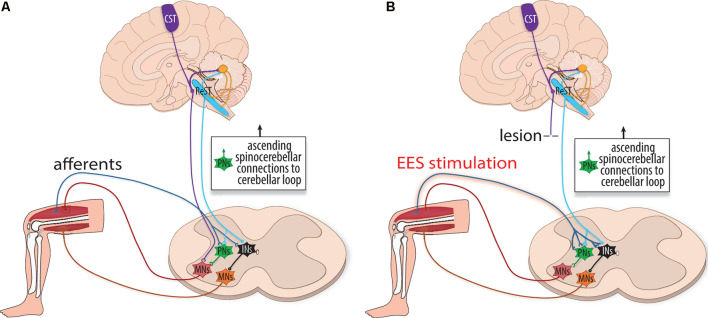
The influence of internal motor copy circuitry during rehabilitation. **(A)** Schematic illustration depicting the internal motor copy, a cerebellar loop that makes a forward prediction of sensory input. It is thought to be primarily performed in the cerebellum and, to a lesser extent, within local spinal circuits. Predictions are compared (and respond) to incoming proprioceptive information to adjust motor actions in real-time before the completion of the movement. Supraspinal networks, including the cortical spinal tract (CST, purple), reticulospinal tract (ReST, blue), and rubrospinal tract (not shown) relay planned motor information to the cerebellum to generate the internal motor copy. Multiple internal motor copies are relayed to the cerebellar loop *via* the Lateral Reticular Nucleus. Simultaneously, sensory information from the periphery is relayed to the cerebellum *via* spinocerebellar tracts. Comparative analyses are performed within this cerebellar loop to communicate what the intended motor command accomplished. Supraspinal fibers converge onto interneurons (INs, black), including (PNs, green), to indirectly excite motoneurons. **(B)** With lesion, fibers from the reticulospinal tract (ReST, blue) may relay cortical commands, as the internal motor copy is unaffected by the lesion and spared and injured PNs can dynamically reorganize after SCI. With EES, input from the ReST spatiotemporally combines with increased activity of group Ia afferents to achieve supra-threshold activation of interneurons as well as indirectly activating motoneurons (MNs, red and orange).

In the absence of pathology, the descending supraspinal control is hypothesized to be involved in three aspects of motor physiology: sensory prediction, real-time adjustments, and motor memory. For example, Straka et al. (2018) discuss how the efference copy, in conjunction with the forward model, predicts the sensory consequences of action so that the central nervous system can routinely ignore the self-generated sensory input produced during the behavior. Azim and Alstermark (2015) used the term internal motor copy to describe the efference copy that is conveyed to the cerebellum to generate predictions of motor actions. The forward model can predict the consequences of a motor command and adjust the output in real-time without having to rely on delayed proprioceptive feedback (Wolpert and Miall, 1996). However, the forward model can also respond to ongoing sensory feedback to refine the accuracy of the outputs (Figure 3).

Post-injury, the descending supraspinal control potentially assumes a principal role in the recovery of locomotion. Asboth et al. (2018) found that residual ReST fibers in a rat contusion study were fundamental to regaining locomotive function. The study involved severe thoracic spine contusions designed to abolish CST fibers, followed by the retraining of lumbar circuits using a strict neurorehabilitation program. Before the injury, the rats were randomly assigned to untrained and trained groups for neurorehabilitation. All the rats that received a rehabilitation program while simultaneously receiving electrochemical neuromodulation regained weight-bearing locomotion, whereas none of the untrained rats were able to produce locomotion (even in the presence of electrochemical neuromodulation). Additionally, the majority of the animals who did not receive any neuromodulation but did receive neurorehabilitation were able to recover locomotion, illustrating that the underlying process was organic in nature. Neuroanatomical tracing confirmed that the contusions interrupted all motor cortex projections to the lumbar segments and that only neurons in the ventral gigantocellular reticular nuclei (vGi), raphe, and the parapyramidal region retained connectivity across the lesion. They concluded that neurorehabilitation and neuromodulation synergistically promoted the reorganization of glutamatergic cortical projections to the vGi and the growth of ReST fibers across the injury, which relayed the cortical commands downstream. Importantly, with the application of Designer Receptors Exclusively Activated by Designer Drugs (DREADDs), they established that these ReST fibers are of little consequence in uninjured animals, and the extensive reorganization of cortico-reticulospinal circuits becomes critical in SCI.

The internal model may have a spinal component functioning independently of the cerebellum. Brownstone et al. (2015) refer to the spinal component in the context of motor learning. They infer that the alpha-motoneurons of the spinal cord may function similarly to the deep cerebellar nuclei by measuring motor command errors during motor learning. The alpha-motoneurons, which produce muscle contraction, receive excitatory sensory information from Ia afferents, inhibitory inputs from Renshaw cells, as well as provide the Renshaw cells with an efferent copy of the commands. They describe the alpha-motoneurons as comparators that assess the discrepancy between motor commands and motor outputs in essence arguing that the cerebellum is not the only CNS structure where forward models are expressed. Takeoka (2020) discusses how the proprioceptive feedback may contribute to intrinsic spinal cord circuitry, and how proprioception helps construct an internal motor command that executes outputs in the event of severed descending pathways. In fact, “movement-specific activation of spinal interneurons and motoneurons combined with intrinsic plasticity of the spinal cord network facilitates learning to walk with limited brain input” (Takeoka, 2020). For example, Forssberg (1979) noted that completely transected cats were able to adjust limb trajectory during the swing phase of locomotion upon encountering an obstacle, thus underlining the existence of an intrinsic spinal network independent of descending input. The conceptual framework of the forward model may thus be separated into two distinctive entities, one confined to the hindbrain and one located in the spinal cord, that are implicated in the recovery of locomotion.

Certain spinal interneurons may contribute to the spinal internal motor circuitry. Bui et al. (2016) demonstrated that dI3 interneurons receive afferent inputs and project onto intermediate and ventral regions of the spinal cord. “The dI3 interneurons are positioned between multimodal sensory input and spinal locomotor circuits, and have a bi-directional relationship with these locomotor circuits, receiving an efference copy of their activity.” They surmised that this spinal microcircuitry is not necessary for normal locomotor activity, but is critical in driving locomotion following transection as it continues to integrate sensory input. In their rodent model, dI3 knockout mice with spinal transection displayed a significant reduction in generating locomotor activity when compared to spinalized control mice. They performed lower thoracic spinal cord transections on both dI3 knockout mice and control mice and then compared locomotor recovery. The performance was quantified using forelimb/hindlimb step ratios, with any forward excursions of the toes (“forward excursions”) counted as steps, and qualitatively assessed using high-speed kinematic video recordings. During recovery, they found that the knockout mice had half the number of steps of the control mice. Furthermore, the knockout mice displayed linear kinematics not at all reflective of locomotion when compared to the control mice using horizontal movement, vertical movement, and joint angles as parameters. As such, dI3 interneurons and the associated circuits could promote sensory-mediated recovery of function in the absence of any descending motor commands, mirroring the automaticity of the proposed descending supraspinal control.

Rehabilitative training with or without EES could provide error correction by either rerouting cerebellar instructions past the injury or at local spinal cord circuit levels. For cerebellar modulation following SCI, descending supraspinal control could be responsive to EES. Lesion studies have found that severed ReST fibers sprout in an ipsilesional manner above the injury to form excitatory boutons, and while descending supraspinal fibers struggle to penetrate the hostile micro-environment of a lesion, they do converge onto interneurons (e.g., PNs) as intermediaries (Flynn et al., 2011; Filli et al., 2014). The reorganization and prioritization of glutamatergic ReST neurons with ancillary projections below the injury could, therefore, relay error adjusted commands following SCI (Fink and Cafferty, 2016; Kim et al., 2017; Asboth et al., 2018). The descending reticulospinal control may facilitate recovery through heterosynaptic plasticity in concordance with EES sensory afferents: the activity of the ReSTs spatiotemporally combines with group Ia afferents to help overcome a threshold needed for interneuronal activation (Figure 3). Therapies utilizing spinal cord stimulation help promote recovery in part by fortifying the spatiotemporal consolidation of activity at the intersection between ReST fibers and group Ia afferents, which in turn stimulate motoneurons.

## Using Chemogenetic Technology to Uncover EES-Induced Mechanisms of Recovery

Although remarkable progress has been made in identifying pathways that support enhanced recovery after SCI with EES, the daunting challenge of pinpointing new and enhanced connections at the cellular and synaptic levels, some of which were illustrated above, remains. Genetic tools may help in this task. Genetic tools enable: (1) targeted, reversible manipulation of specific pathways and neuronal subpopulations; (2) labeling of precisely which neurons have been modulated (not definitively known with electrical stimulation); and (3) identification and subsequent tracing of secondary circuits that have been influenced. Multiple genetically encoded tools for remote control of the nervous system now exist on timescales ranging from milliseconds (e.g., optogenetics) to hours (e.g., chemogenetics), as well as viral and transgenic methods to restrict their expression to defined neural groups or phenotypes (e.g., motor, proprioceptive, or nociceptive; Towne et al., 2013; Iyer et al., 2016).

**Figure 4:**
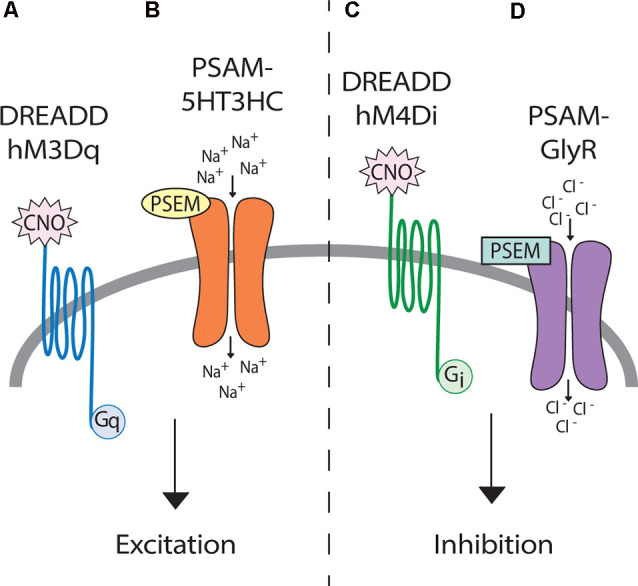
Genetic tools for afferent modulation. (Left) Both DREADD hM3Dq **(A)** and PSAM-5HT3HC **(B)** can make neurons more excitable through depolarization. (Right) Both DREADD hM4Di **(C)** and PSAM-GlyR **(D)** can inhibit neuronal activity through hyperpolarization. Upon clozapine-*N*-oxide (CNO) binding, DREADDs activate G-protein coupled signaling cascades, which ultimately change cellular membrane potentials. In contrast, upon binding by PSEMs, PSAMs directly open ion channels allowing the influx of sodium (excitatory) or chloride (inhibitory).

Unlike optogenetics, chemogenetics provides the advantage of not requiring a tether, thus neuromodulation can be studied in freely behaving animals. Chemogenetic technology has the potential to achieve behaviorally relevant excitation or inhibition of neural phenotypes upon administration of an actuator molecule (either an injected drug or given food item). DREADDs are perhaps the most well established chemogenetic tool for neuromodulation and work by manipulating G-protein coupled pathways (Figures 4A,C; Armbruster et al., 2007; Roth, 2016). Relevant to neural dysfunction and repair, Jaiswal and English (2017) found that activation of motoneurons with excitatory DREADDs in a rat peripheral nerve injury model could improve functional recovery. In a rat contusion model of SCI, targeted DREADDs-silencing was used to identify glutamatergic neurons of the ventral gigantocellular reticular and vestibular nuclei as responsible for transmitting a cortical command to lumbar neurons for trunk stability and patterned movements (Asboth et al., 2018). In a mouse staggered bilateral hemisection model of SCI, DREADDs hyperpolarization of inhibitory interneurons identified these interneurons as the limiting factor preventing supraspinal commands from propagating into relay circuits (and putatively lumbar CPG centers) after injury (Chen et al., 2018). Although the mechanism of action of DREADDs agonist clozapine-*N*-oxide (CNO) has been questioned (Gomez et al., 2017; Mahler and Aston-Jones, 2018), experimental designs with between-subject controls can make CNO (3–5 mg/kg) a suitable DREADD agonist (Jendryka et al., 2019). Another chemogenetic tool, Pharmacologically Selective Actuator Modules and their Effector Molecules (PSAMs/PSEMs), works *via* directly opening ion channels in neurons (Figures 4B,D) for robust neural excitation and silencing (Magnus et al., 2011). A recently developed PSAM, PSAM^4^-GlyR, is an ultrapotent chemogenetic receptor for varenicline, an FDA-approved smoking cessation drug. PSAM^4^-GlyR overcomes limitations from using traditional PSEMs, such as short clearance times (30–60 m) and low-micromolar potency, making it highly applicable for *in vivo* studies (Magnus et al., 2019). The control of specific neurons *via* administration of a drug, and subsequent neuronal tracing capability, make chemogenetics an important tool for modulating circuits to understand molecular mechanisms of plasticity.

Importantly, chemogenetic manipulation of *afferent* activity holds promise to uncovering molecular and circuit mechanisms of EES-induced recovery from SCI. For example, if chemogenetics was restricted to, and altered excitability of, afferents activated by EES (medium and large diameter afferents within the posterior roots) in SCI models, the neural circuit changes that were induced by these afferents could be quantified in postmortem histological analyses. In addition to tracing modulated pathways and definitive knowledge of which afferents were affected, it opens the door to combinatorial modulation of subsets of types of afferents (e.g., excite only proprioceptors without affecting exteroceptors, whilst inhibiting nociceptors). As with EES, locomotor changes from afferent excitation (or *inhibition* with chemogenetic tools) can be identified using assays such as high-speed kinematics. However, the main strength of chemogenetic tools lies in the unique advantage of identification of plastic mechanisms that occur during recovery from SCI, a unique ability that EES cannot replicate.

## Conclusion

EES is a potentially effective therapy to enhance sensorimotor recovery following SCI. However, the exact mechanisms underlying recovery remain elusive. This review identifies several plasticity mechanisms that may be evoked by EES through the activation of peripheral afferents. Resultant recovery is likely due to local lumbar, propriospinal, and internal models acting together synergistically. While the propriospinal network and the descending reticulospinal command are putatively most contributive to recovery from anatomically incomplete lesions, recovery from complete lesions is likely due to local lumbar circuit plasticity driven by afferent input. The identification of these mechanisms of plasticity will likely be accelerated by genetic tools for afferent modulation.

## Author Contributions

All authors contributed to the conceptualization and writing. JE led the writing process. KK led the artwork and figures. RS led the writing on the introduction, history, and internal motor copy. ML, GS, and AS contributed to the conceptualization, writing, and editing.

## Conflict of Interest

The authors declare that the research was conducted in the absence of any commercial or financial relationships that could be construed as a potential conflict of interest.
